# A Simulation Approach for the Spatial Testing of Migration Theories

**DOI:** 10.1007/s10680-026-09770-0

**Published:** 2026-03-06

**Authors:** Micol Matilde Morellini

**Affiliations:** 1 Nuffield College, University of Oxford, New Road, OX1 1NF Oxford, UK; 2 Department of Sociology and Leverhulme Centre for Demographic Science, University of Oxford, 42-43 Park End Street, OX1 1JD Oxford, United Kingdom; 3 Department of Sociology, University of Zurich, Zurich, Switzerland Andreasstrasse 15, 8050

**Keywords:** Migration theory, Spatial analysis, Simulation methods, Model evaluation, Migration systems

## Abstract

Migration research has long been divided between studies of drivers, which focus on the factors shaping migration flows, and studies of patterns, which describe how these flows are organised across space. Theories of migration typically identify and operationalise drivers, but are often less explicit about patterns. As a result, migration theories are usually evaluated using goodness-of-fit measures that assess explanatory power but pay limited attention to spatial accuracy. This article addresses this limitation by introducing a simulation-based procedure to evaluate the spatial accuracy of migration theories. Starting from an observed system of origin–destination migration flows, the procedure generates synthetic systems that reflect the spatial outcomes implied by a given theory. These synthetic migration systems are then compared to the observed case to assess spatial accuracy. The procedure is applied to intra-European migration flows from 2002 to 2021 and illustrated using two long-standing migration theories: the gravity model and migration systems theory. Both theories achieve high explanatory power under conventional goodness-of-fit metrics, and migration systems theory performs better overall. However, the empirical analysis shows that both theories fail to reproduce important spatial features of the European context, including the high level of reciprocity of flows and the observed migration profiles of Eastern and Northern European countries. These findings highlight how strong statistical fit does not imply accurate spatial representation. Evaluating migration theories through their implied spatial outcomes provides new insights into their limitations and offers a complementary and integrative tool for migration research.

## Introduction

In recent years, the study of human migration has expanded into a diverse and interdisciplinary field, reflecting the complexity of migration as a social phenomenon (Brettell and Hollifield, 2022; Levy et al., 2020; Scholten et al., 2022). Migration complexity captures how migration flows are driven by factors operating at multiple levels (micro, meso, and macro drivers) and simultaneously exhibit empirical regularities in their spatial organisation, known as migration patterns. This inherent complexity has contributed to substantial theoretical fragmentation within migration studies, where paradigms on migration drivers and patterns have developed largely in parallel (Arango, 2000; Garip, 2012; Massey et al., 1993; Massey, 2019 ).

Migration theories typically identify which migration drivers may influence migration flows. At the same time, theories carry implicit expectations about how migration should be organised across space: for instance, whether flows should concentrate within a small number of corridors or exhibit strong directional asymmetries. Yet, such spatial implications are rarely articulated explicitly or subjected to empirical evaluation. One fundamental reason is methodological: while migration drivers are routinely operationalised as predictors in explanatory models, there are few tools for reconnecting drivers to the migration patterns they are presumed to generate. As a result, empirical research has largely assessed theories through conventional goodness-of-fit metrics, which indicate whether predictors help explain flows but provide little insight into whether the resulting spatial configurations resemble those observed in real-world migration cases. Spatial features may be included as control variables, but they are seldom treated as substantive outcomes in their own right, leaving unresolved whether competing migration theories serve as spatially accurate representations of migration processes.

This article offers an alternative approach. By treating migration patterns as emergent outcomes of theory-guided migration drivers, it evaluates whether drivers produce spatial features consistent with observed data. In doing so, the article introduces a methodological framework to evaluate migration theories based not only on their explanatory power and significance of drivers, but also on their spatial accuracy. This is in contrast with standard spatial econometric approaches, where spatial dependencies are treated as explanatory or control inputs; instead, space is regarded as a dimension of interest that theories should be able to reproduce. The proposed method has two main components. First, it estimates bilateral migration flows using regression models that incorporate drivers derived from established migration theories. Second, it simulates counterfactual migration systems under the specified theoretical assumptions and compares simulated and observed spatial features, while also quantifying uncertainty.

To demonstrate this approach, I apply it to international migration within Europe between 2002 and 2021. Europe provides an ideal empirical setting because it constitutes a relatively controlled institutional environment with extensive, harmonised bilateral migration data. Crucially, the European Union’s (EU) freedom of movement regime enables high levels of mobility within its greater borders, creating a quasi-natural experiment in institutionally facilitated international migration. At the same time, substantial cross-country variation in migration volumes has attracted extensive theoretical attention, making Europe a natural laboratory for evaluating how different migration drivers may generate different patterns (Raymer, 2016). In this context, I examine two prominent theories of international migration: the gravity model (Ramos and Suriñach, 2017) and Migration Systems Theory (MST; DeWaard et al. 2012).

The intra-European application shows that, while both the gravity and MST models perform well in terms of conventional explanatory power (eg adjusted $R^2$), they fall short in reproducing several spatial features of the observed migration system. In particular, the two theories capture the concentration of intra-European migration flows but struggle to reproduce their reciprocity and the specific migration profiles of Eastern and Northern European countries, both in terms of inflows and corridor structures. These findings highlight the limits of conventional goodness-of-fit metrics and underscore the value of spatially informed evaluation techniques.

The article makes three main contributions. First, it reconnects the study of migration drivers with that of migration patterns by treating the latter as emergent features of the former. Second, it introduces a method to evaluate migration theories based on their ability to generate empirically plausible spatial patterns. Third, it offers a scalable and generalisable framework for theory-testing that can be applied across different migration systems, both international and internal.

The remainder of the article is organised as follows. In Sect. 2, I review the theoretical and methodological background motivating the proposed method. In Sect. 3, I present the method in detail. In Sect. 4, I apply and illustrate it in the context of intra-European migration flows. Finally, in Sect. 5 I reflect on the findings and their limitations, outlining implications for future research.

## Theoretical and Methodological Background

Once considered demography’s ‘stepchild’ (Kirk, 1960), the study of human migration has evolved into a broad research field covering a large number of subtopics and recognising the contribution of different disciplines, including demography, geography, population ecology, sociology, and economics (Brettell and Hollifield, 2022; Levy et al., 2020; Scholten et al., 2022). Yet, these disciplines have often developed in parallel rather than in dialogue, resulting in what has been described as academic ‘tribalism’ or ‘sectarianism’ (Natter and Welfens, 2024, p. 18).

One consequence of this fragmentation is a persistent divide between research traditions that focus primarily on migration *patterns* and those that focus primarily on migration *drivers*. Work in human geography, spatial demography, and population ecology has tended to emphasise migration patterns and the spatial organisation of migration flows (Héran, 2022; Price, 2022). By contrast, scholarship in sociology, social demography, and economics has predominantly focused on the drivers of migration, identifying the factors that motivate, enable, or constrain individual and aggregate mobility (Czaika and Reinprecht, 2022; FitzGerald, 2022; Martin, 2022).

Although these two strands are conceptually complementary, they are rarely implemented together. Driver-oriented research typically evaluates predictors linked to theoretical mechanisms, but not their spatial accuracy; conversely, pattern-oriented research analyses the emergent system-level regularities produced by migration flows, without investigating their social and behavioural origins. This section outlines each tradition in turn before introducing a framework that bridges them by treating spatial patterns as emergent outcomes of migration drivers.

### Conceptualising Migration Patterns

By connecting physical locations, migration flows serve as spatial linkages. These spatial linkages represent strong empirical regularities that can be mapped, measured, and described in what are usually known as *migration patterns*. Migration patterns thus describe the characteristics of migration ‘systems’, that is, configurations consisting of locations and the flows that connect them (Bakewell, 2024; Boyle et al., 1998; DeWaard and Ha, 2019; Raymer et al. 2019). Migration systems emphasise the spatial organisation, or geographical structure, of migration and the aggregate (systemic) level of analysis (Bakewell, 2024); as such, they are distinct from migration systems *theory*, which is presented in greater detail in Sect. 2.2^1^.

To analyse migration systems and their patterns, researchers have developed a host of system-wide descriptive indices. Among these, indices of migration *concentration*, also known as spatial focusing, measure how unevenly migration flows are distributed within a system; in other words, the extent to which migration flows concentrate within a few origin–destination corridors (Czaika and de Haas, 2014; Plane and Mulligan, 1997; Rogers and Sweeney, 1998 ). Indices of migration *reciprocity*, also known as migration effectiveness, measure to what extent a migration flow from a location *i* to a location *j* is occurring also in the opposite direction, from *j* to *i* (Charles-Edwards et al. 2025; Huang and Butts, 2023; Leal and Harder, 2023; McMillan, 2024).

As empirical regularities and ‘structural imprints’, migration patterns represent the aggregate manifestations of underlying migration processes (DeWaard and Ha, 2019). This idea forms the basis of the methodology proposed in this article, which evaluates theories in terms of the spatial patterns they imply.

### Conceptualising Migration Drivers

In the case of international migration, drivers refer to the factors that motivate or constrain changes in residence or moves away from one’s country of origin (conventionally, of birth or nationality). Migration drivers include micro-level factors, such as individual aspirations, motivations, and capabilities, meso-level factors, such as interpersonal networks and other intervening factors, and macro-level factors such as geographic, historical, and politico-institutional conditions (Czaika and Reinprecht, 2022; de Haas, 2021).

Traditionally, migration theories are operationalised as sets of migration drivers, that are then used as predictors of migration flows (the outcome variable) in explanatory models (Garip, 2012). One of the classic models exemplifying this approach is the gravity model, first developed by Ravenstein (1885, 1889). The gravity model posits that migration between two locations increases with population size and decreases with geographic distance between them. Though simple, the gravity model has proven durable and remains widely used across demography, economics, and sociology (see Botezat and Ramos, 2020; Cohen et al. 2008; Poot et al. 2016; Ramos, 2016; Ramos and Suriñach, 2017).

A pivotal contribution in the operationalisation of migration theories is provided by Massey et al. (1993). Massey et al. survey eight major theories of migration: the neoclassical economic model, the new economics of labour migration, dual labour market theory, world systems theory, network theory, institutional theory, cumulative causation, and MST. In all cases, the authors ‘translate’ the abstract notions and mechanisms of migration theories into measurable and testable drivers. Further, the authors identify MST as the most comprehensive framework, as it synthesises mechanisms from many of the preceding theories and integrates a wide array of geographic, economic, institutional, historical, and socio-cultural linkages to explain the persistence and structure of bilateral migration flows. In practice, MST is operationalised as a wide set of predictors, including: (i) geographic predictors such as distance and contiguity, akin to gravity models; (ii) economic differentials between origins and destinations, drawing from neoclassical and new economics perspectives; (iii) prior migration flows and stocks, consistent with theories of network migration and cumulative causation; and (iv) predictors stemming from world-systems and institutional theories, such as colonial and other historical relationships between locations, shared language, and institutional membership (DeWaard et al., 2012; Leal and Harder, 2023, Massey et al. 1993).

While many migration theories are potentially relevant for empirical testing, this article focuses on the gravity model and MST because they represent two ends of a spectrum of theoretical sophistication. The gravity model is parsimonious and centres on a small number of macro-level predictors, most notably population size and geographic distance, whereas MST is more elaborate and integrates a broader set of economic, institutional, historical, and relational factors. Notably, both theories assign primary importance to macro-level drivers and operate at the same analytical scale, making them directly comparable within a unified modelling framework. At the same time, they differ in the extent to which they articulate expectations about the spatial organisation of migration systems. In the gravity model, migration patterns emerge implicitly from dyadic and largely symmetric attributes: flows are shaped by distance and size, but the theory itself makes few explicit claims about the resulting system-level spatial organisation. Considering its main drivers, we might conclude that gravity would imply relatively even and reciprocal patterns of exchange across space than other theories, though not necessarily fully symmetric ones. MST, by contrast, is more explicit in its spatial expectations. It conceptualises migration systems as structured by hierarchical and historically embedded relations, anticipating core–periphery configurations characterised by concentrated flows, directional asymmetries, and low reciprocity (Leal and Harder, 2023; Windzio, 2018). These differences in how explicitly spatial structure is theorised make gravity and MST particularly well-suited for comparative evaluation. By assessing not only how well each model explains bilateral flows, but also how accurately it reproduces observed spatial patterns, the analysis moves beyond standard goodness-of-fit metrics and toward a more comprehensive, spatially explicit assessment of theoretical validity.

### Closing the Gap Between Migration Patterns and Drivers

The contrasting spatial expectations of gravity and MST highlight a broader methodological challenge: despite clear theoretical links between migration drivers and patterns, most empirical approaches inspect them separately. While recent scholarship has called for more spatially explicit approaches to migration modelling, existing quantitative frameworks still fall short of integrating these dimensions in a unified way (Czaika et al. 2025; Hoffmann et al. 2021; Matthews et al. 2021; Ton et al. 2024).

Prominent attempts to bring space into empirical migration analyses can be found in the literature on spatial econometric models, which incorporate spatial dependence through spatially lagged predictors or correlated error structures (Elhorst, 2013). While these models account for autocorrelation and improve statistical inference, they conceptualise space primarily as a predictor, correction term, or mechanical dependence structure. Spatial components are introduced to reduce bias or model spillovers, not to analyse spatial structure as the outcome of interest. Consequently, the statistical significance of individual predictors tells us little about a model’s spatial accuracy: in the migration case, they do not tell us whether a given combination of drivers, even when well-specified and significant, can reproduce the spatial configuration of observed migration systems. For the same reason, existing approaches offer limited tools for assessing uncertainty around spatial accuracy, making it difficult to evaluate spatial robustness (Matthews et al., 2021).

To address these gaps, this article proposes a simulation-based method that treats migration patterns as emergent outcomes of theoretical models, rather than as nuisance terms or variables to be controlled for. By (i) estimating flows under competing sets of drivers; (ii) simulating full counterfactual migration systems; (iii) comparing their spatial patterns to those of the observed system; and (iv) incorporating uncertainty, the method directly evaluates the spatial accuracy of migration theories. In doing so, it responds to calls for methodologies that ‘bring together spatial thinking and demographic thinking’ (Raymer, in Matthews et al., 2021, p. 5) and provides a unified framework for adjudicating between alternative explanations on both statistical and spatial grounds, thereby allowing for a more comprehensive assessment of which theories best reproduce the architecture of real-world migration systems.

### Simulation Approaches in Demography

The simulation-based spatial tests developed in this article build on advances in simulation modelling and random networks. Over the past twenty years, both simulation and network approaches have become increasingly widespread in demography and migration studies (Bilecen et al., 2018; Drouhot et al., 2023; Kashyap and Zagheni, 2023). Simulation approaches are usually divided between micro-level simulations, which include Agent-Based Models (ABMs) and microsimulations, and macro-level simulations, which include system dynamics and complex networks (Kashyap and Villavicencio, 2016; Margetts and Dorobantu, 2023; Raftery, 2000). As the specific differences between micro and macro simulation approaches are beyond the scope of this article, the remainder will discuss simulation strategies more broadly.

The relevant contribution of both micro- and macro-level simulations to the proposed method is that they detect theoretical mechanisms underlying emergent patterns, based on individual (micro) or system (macro) behaviours (Drouhot et al., 2023; Klabunde and Willekens, 2016). In practice, simulation models in migration research combine a small number of core ingredients. First, they define a set of entities—such as individuals, households, or locations—and a finite set of states these entities can occupy (eg residence in a given location, migrant *vs* non-migrant status). Second, transition rules are specified to regulate how entities move between states over time. These rules are typically probabilistic and derived from empirical models, stylised behavioural assumptions, or secondary data. Simulation approaches can thus be seen as multilevel and multistate, as agents and systems are allowed to transition between various states (Bijak et al., 2018). For example, at the micro-level, ABMs have been used to explore how environmental change shapes rural–urban and international migration decisions (Kniveton et al., 2011) and how social networks sustain and amplify migration streams over time (Klabunde and Willekens, 2016); at the macro level, simulations have been applied to examine how migration routes emerge and stabilise over time (Bijak, 2022) and how internal mobility flows may respond to economic and policy shocks (Huang and Butts, 2023, 2024, ).

Both micro- and macro-level simulations can be used to generate synthetic counterfactual and ‘what if’ scenarios, which can be particularly valuable for exploring causal relationships in demography, where experimental designs such as randomised controlled trials may be infeasible or ethically inappropriate (Kashyap and Zagheni, 2023). For the same reason, simulations can be used for hypothesis testing. While this aspect remains somewhat underutilised in demography and migration studies, a broader literature in sociology, as detailed in the next subsection, motivates the implementation of simulations for theory-testing in the method proposed here.

### Simulations for Hypothesis Testing

One of the most important research pieces establishing the connection between simulations and hypothesis testing is by King et al. (2000). In their article, King et al. introduce simulations as a powerful tool to extract key insights from quantitative data and make empirical findings accessible to non-technical audiences. The authors posit that social scientists tend to focus too narrowly on technical terms such as *p* values and statistical significance, limiting the wider interpretability of findings. By contrast, simulations enable researchers to present results as precise estimates of real-world quantities and counterfactuals. King et al. argue that the use of simulations for hypothesis testing offers three major benefits. First, it allows researchers to extract theoretically meaningful quantities from statistical models. Second, it clarifies, also visually, the uncertainty surrounding estimates without referring to technical terms. Lastly, it makes complex findings accessible to a broader audience without requiring specialised statistical knowledge.

In sociology, King et al.’s simulation-based workflow has inspired applications across several domains. For example, Alon and Gelbgiser (2011) use post-estimation simulations to quantify how horizontal sex segregation across fields of study contributes to gender gaps in college completion. Block (2023) adapts the simulation framework to networks to examine how individual transitions in occupations aggregate into patterns of gender segregation in the labour market. Similarly, Huang and Butts (2023, 2024) employ network-based simulations to examine how diverging tendencies towards internal migration or immobility can produce spatial and social segregation at the aggregate level of US counties. Across these studies, simulations serve primarily to derive interpretable quantities from statistical models, explore counterfactual scenarios, or examine how assumed behavioural rules translate into macro-level distributions.

The approach proposed in the present article draws on and extends these strategies. Like previous work, it uses simulations to generate counterfactual systems under alternative assumptions. However, rather than simulating behavioural outcomes conditional on a fixed network or population configuration, the method simulates entire bilateral migration systems implied by competing theoretical models, explicitly incorporating simulation-based uncertainty around spatial accuracy. This allows researchers to assess whether the spatial structures produced by a given migration theory align with those observed in the data, broadening simulation-based inference for the purpose of spatial theory-testing.

## Proposed Methodology: A Spatial Test for Migration Analysis

**Fig. 1:**
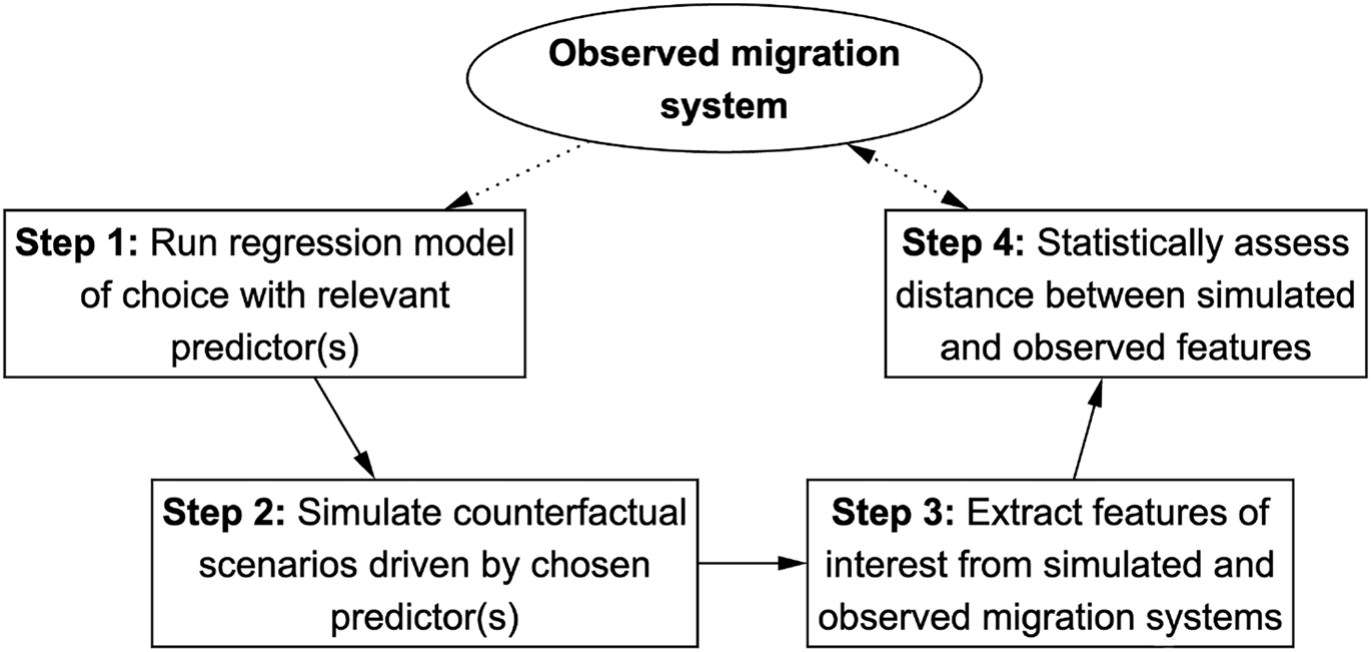
Diagram of the porposed simulation-based procedure.

The method developed here evaluates the spatial accuracy of migration models by linking migration drivers to observed patterns. It consists of four main steps, summarised in Fig. 1. These steps are designed to be flexible and adaptable, with the methodological contribution lying primarily in the *sequence* of operations, not in the specific modelling choices. This ensures applicability across a wide range of migration contexts and data structures.

To illustrate the approach, I present one possible implementation, which relies on: a regression-based model (Step 1), a computationally efficient simulation technique (Step 2), established spatial indices from migration research (Step 3), and Mahalanobis distance for model evaluation (Step 4). Notably, the simulation technique used here is designed for scalability: in the empirical application in Sect. 4, millions of individuals across 31 European countries over 20 years are simulated in seconds on a standard workstation using the statistical software R  (version 4.3.2; R Core Team, 2023).

### Step 1: Model Specification

The first step of the procedure requires estimating a statistical model of migration, in which bilateral flows are explained as a function of theory-informed drivers. As detailed in Sect. 2.2, different theoretical frameworks can be operationalised as alternative sets of predictor variables. An explanatory model of migration flows could then take the following functional form:

1 $$\begin{aligned} y_{ij} = \beta \boldsymbol{x}_{ij} + \varepsilon _{ij}, \end{aligned}$$

where the dependent variable, $y_{ij}$, is the migration flow from an origin *i* to a destination *j*, estimated as a function of predictor variables, contained in the vector $\boldsymbol{x}_{ij}$, plus an error term, $\varepsilon _{ij}$. The model’s coefficients are included as $\beta$.

Step 1 translates theoretical assumptions into testable model inputs. Each set of predictors represents not only a claim about what drives migration flows, but also an implicit prediction about what the resulting spatial patterns should look like. It is recommended that this step accounts for data interdependencies that are common in migration research. Estimators such as Poisson Pseudo-Maximum Likelihood (PPML) with origin and destination fixed effects (Santos Silva and Tenreyro, 2006), or network regressions with Quadratic Assignment Procedure (QAP; Barnett and Nam, 2024; Plotnikova and Ulceluse, 2022) can address these dependencies.

### Step 2

Step 2 implements simulation techniques to generate alternative, theory-guided migration scenarios. To ensure direct comparability with the migration drivers specified in Step 1, the simulations are based on the fitted values from the regression model in Equation (1). Following the logic of simulation-based inference introduced by King et al. (2000), uncertainty around the estimated migration flows is propagated into the spatial domain by repeatedly simulating complete migration systems implied by the fitted model.

In practice, simulations are carried out at the level of bilateral flows. For each origin–destination pair (*i*, *j*) and year, a fitted flow ${\hat{y}}_{ij}$ and its associated confidence interval are obtained from the regression model. Assuming approximate normality of the sampling distribution of the fitted values, the width of the confidence interval is used to recover a standard deviation for each dyad. Simulated flows are then drawn independently for each dyad from a truncated normal distribution:

2 $$\begin{aligned} y_{ij}^* \sim \mathcal{T}\mathcal{N}\!\left( {\hat{y}}_{ij},\, \sigma _{ij}^2;\, a_{ij},\, b_{ij}\right) , \end{aligned}$$

where $\sigma _{ij}$ is derived from the confidence interval bounds $(a_{ij}, b_{ij})$. Truncation ensures that simulated flows remain within empirically plausible ranges and prevents the generation of negative migration values.

Each draw across all dyads constitutes a single simulated migration system, represented as an origin–destination flow matrix. To ensure close correspondence with the observed migration system, simulated flows can be calibrated so that total outflows from each origin match the observed outflows in the corresponding year. This row-wise scaling preserves the set of origins and destinations, prevents the appearance of migration corridors that are not observed in the data, and ensures that the aggregate volume of migration in each simulated system matches that of the observed system.

Repeating this procedure yields an ensemble of counterfactual migration systems implied by the fitted model. Rather than producing point predictions for individual flows, the simulation strategy generates a distribution of possible migration systems consistent with the estimated drivers and their uncertainty. This ensemble provides a sampling distribution for both flow-level quantities and system-level spatial features, which are used in Step 4 to assess how unusual the observed migration system is relative to what a given theoretical model would imply.

Crucially, the simulation strategy adopted here is computationally efficient and well suited for testing theories that emphasise macro-level drivers, such as the gravity model and MST. A limitation of this approach is that dyadic flows are simulated independently and do not explicitly model individual destination-choice behaviour. While this makes the approach scalable to large migration systems, it abstracts from micro-level decision processes. In principle, Step 2 could be implemented using individual-level simulation strategies, such as microsimulations or ABMs, allowing for agentic destination choice, albeit at a substantially higher computational cost.

### Step 3

Step 3 extracts spatial features of interest from the simulated migration systems, enabling direct comparison with observed data. Here, I rely on four migration indices, which are described below, but alternative or additional indices could be employed to describe migration patterns.

In most cases, and all those included here, migration indices are computed using matrix notation, where a migration system, $\boldsymbol{M}$, is a matrix with rows representing the origins, and columns the destinations, so that an element $m_{ij}$ will report the total number of people moving from location *i* to location *j*. If, between two locations, no migration flow is observed in that specific direction, the corresponding matrix element will be equal to zero. By nature of most migration analyses, where the focus is on individuals who decide to relocate, the diagonal of the origin–destination matrix is usually set equal to structural zeroes (Raymer and Rogers, 2007). In the case of longitudinal data, a migration matrix, its elements, and any descriptive index may refer to a specific time period, *t*. For clarity and simplicity, this temporal subscript is not reported in the following mathematical notations and formulae.

#### Measures of Migration Concentration

Indices of migration concentration quantify how unevenly migration flows are distributed across a system: whether flows are spread evenly across many corridors or concentrated within a few dominant ones. Because different indices weight different parts of the flow distribution differently, three complementary measures are used here to triangulate spatial concentration: the Average Coefficient of Variation (ACV), the Gini Index, and the Migration Inequality Index (Rogers and Raymer, 1998).

**Aggregate Coefficient of Variation (ACV)**

The ACV, first applied to migration by Rogers and Sweeney (1998) and Rogers and Raymer (1998), measures relative dispersion as the ratio of the standard deviation to the mean. Applied to a migration matrix $\textbf{M}$ with *n* locations, it is:

3 $$\begin{aligned} \text {ACV} = \sum _{i=1}^{n} \left( \frac{\sqrt{\frac{1}{n-1} \sum _{j \ne i} (m_{ij}-{\bar{m}}_i)^2}}{{\bar{m}}_i} \right) \cdot \frac{\sum _{j \ne i} m_{ij}}{\sum _{i=1}^{n} \sum _{j \ne i} m_{ij}}, \end{aligned}$$

where $m_{ij}$ is the flow from *i* to *j* and ${\bar{m}}_i$ is the mean outflow from *i*. The ACV has no upper bound and is highly sensitive to the presence of one or two disproportionately large corridors, also known as primacy. Rogers and Raymer (1998, pp. 64–66) emphasise that this sensitivity helps distinguish systems where a few flows dominate from those where concentration is more evenly distributed.

**Gini Index**

The Gini Index of migration measures concentration across all bilateral corridors and ranges from zero (no concentration) to one (maximal concentration; Bell et al. 2002; Plane and Mulligan, 1997):

4 $$\begin{aligned} \text {Gini} = \frac{\sum _{i=1}^n \sum _{j \ne i} \sum _{k=1}^n \sum _{l \ne k} | m_{ij} - m_{kl} |}{(2n(n-1)-1) \sum _{i=1}^n \sum _{j \ne i} m_{ij}}, \end{aligned}$$

where each flow $m_{ij}$ is compared to every other flow $m_{kl}$ in the system. The denominator normalises the index so that it ranges between zero and one. Compared to the ACV, the Gini Index is less influenced by extreme values and more sensitive to changes in the middle of the flow distribution (Rogers and Raymer, 1998, p. 65). This makes it a useful complement for assessing overall inequality without undue weighting toward the largest corridor(s).

**Migration Inequality Index**

The Migration Inequality Index measures the deviation of observed flows from a hypothetical uniform distribution, in which all corridors would contain the same number of migrants (Bell et al., 2002). It ranges from zero (uniform distribution; all migration corridors equally used) to one (maximal concentration; only one corridor used):

5 $$\begin{aligned} \text {Inequality} = \frac{ \sum _{i=1}^n \sum _{j \ne i} | m_{ij} - m'_{ij} | }{2 \sum _{i=1}^n \sum _{j\ne i} m_{ij}}, \end{aligned}$$

where $m'_{ij}$ represents the expected flow under a uniform distribution. While conceptually similar to the Gini, this index is more strongly affected by outliers and large deviations from the expected flow distribution, and therefore sits between the ACV and Gini.

#### Measures of Migration Reciprocity

Indices of migration reciprocity measure whether and to what extent migration flows are symmetric: in other words, whether flows occur in both directions of a migration corridor, from location *i* to location *j* and from location *j* to location *i* (Huang and Butts, 2023; Krivitsky, 2012; McMillan, 2024; Squartini et al. 2013). Following Krivitsky (2012), the reciprocity of a migration system can be computed by comparing the two flows in each corridor and reducing them to a single value using their minimum, maximum, or geometric mean, before aggregating across all corridors.

In Sect. 4 of this article, I use the minimum specification, which focuses on the smaller of the two directional flows in each corridor:

6 $$\begin{aligned} \text {Reciprocity} = \frac{\sum _{i=1}^n \sum _{j \ne i} \min (m_{ij}, m_{ji})}{\sum _{i=1}^n \sum _{j \ne i} m_{ij}}. \end{aligned}$$

The denominator normalises the measure so that it ranges between zero and one. Substantively, the numerator counts the number of migrants who can be ‘matched’ with a migrant moving in the opposite direction in the same corridor, while the denominator is the total number of migrants in the system. The index can therefore be interpreted as the share of migrants who are part of reciprocal exchanges: for example, a value of 0.7 indicates that 70% of all migrants belong to corridors where flows occur in both directions. Values near zero indicate highly asymmetric systems dominated by one-way flows, whereas values near one indicate highly reciprocal systems in which most corridors are approximately balanced.

### Step 4

The simulated migration systems produced in Step 2 represent theory-guided counterfactuals: they show what the spatial structure of migration would look like if flows were driven solely by the predictors specified in Step 1. Step 4 evaluates the spatial accuracy of these theoretical models by comparing the observed migration system with the ensemble of simulated systems.

Building on the logic of random networks and Conditional Uniform Graph (CUG) tests, Step 4 repeats the sampling process described in Step 2 to generate a reference distribution of spatial features under each theoretical model. The comparison between observed and simulated features can therefore be interpreted as a spatial test of goodness-of-fit. This approach is analogous in spirit to simulation-based assessments of Exponential Random Graph Models (ERGM; Lusher et al., 2012), but allows for more calibrated modelling of bilateral migration flows. Because each spatial characteristic is analysed across all simulated systems, the method evaluates not a single point estimate but the full distribution of spatial outcomes implied by each theoretical model.

Model evaluation proceeds in two complementary ways. First, visual inspection of the simulated distributions provides an intuitive assessment of how well each model reproduces the observed spatial features; graphical diagnostics are particularly useful for identifying patterns of model misfit (see King et al., 2000). Second, statistical testing can be carried out using distance measures that quantify discrepancies between observed and simulated systems. Here, I rely on the Mahalanobis distance, which has been used extensively to evaluate the accuracy of statistical network models (Lospinoso and Snijders, 2019). In the migration context, Mahalanobis distance can be applied either to migration indices (eg concentration or reciprocity) or to full vectors of flows for each origin or destination. This allows us to identify not only which spatial features are poorly captured by a model, but also which specific locations contribute most to the misfit.

Formally, for each location or migration corridor, we compare an observed flow vector, $\boldsymbol{x}$, which can represent inflows, outflows, or both, to the distribution of simulated flow vectors $(s = 1,2,\ldots ,S)$. Let $\boldsymbol{\mu }$ and $\mathbf {\Sigma }$ denote the mean and covariance matrix of the simulated vectors, the Mahalanobis distance is then:

7 $$\begin{aligned} D^2 = (\boldsymbol{x} - \boldsymbol{\mu })^T \mathbf {\Sigma }^{-1} (\boldsymbol{x} - \boldsymbol{\mu }). \end{aligned}$$

By incorporating the inverse covariance matrix, Mahalanobis distance accounts for dependencies between origin–destination pairs, providing a multivariate measure of how unusual the observed flows are relative to the simulated distribution. Large values of $D^2$ indicate that the observed flows differ substantially from what the model predicts, whereas values near zero indicate close agreement. In longitudinal analyses, this metric can also be tracked to assess whether spatial accuracy improves or deteriorates over time.

## Application: International Migration in Europe, 2002–2021

To demonstrate how the proposed four-step procedure can be used in practice, I apply it to international migration in 31 European countries from 2002 to 2021 2. The European case is particularly well-suited for testing the proposed spatial tests for four reasons. First, Europe offers a relatively controlled institutional environment with high levels of cross-border mobility. The EU Treaty of Maastricht (1992) and the Free Movement Directive (in force since 2004) grant citizens of EU member states reciprocal rights to move, reside, and work across borders, creating a quasi-natural experiment in free international migration within the EU’s wider boundaries (European Commission, 2023; Maciejewski, 2024; Marzocchi, 2024). Further bilateral agreements have been established with non-EU countries in the continent, including the Schengen Agreement (first established in 1985) and the Agreement on the Free Movement of Persons (AFMP) between Switzerland and the EU (2002). This symmetric mobility rights regime is especially relevant for assessing spatial features such as reciprocity and corridor structures. Second, Europe is an exceptionally data-rich context (Raymer, 2016). Nordic countries in particular provide high-quality register data, and migration inflows and outflows for most countries in the region are recorded by national statistical offices as well as supranational institutions such as Eurostat (Dańko et al., 2024). The availability of multiple sources facilitates internal consistency checks between data provided by sending and receiving countries, supporting the construction of harmonised bilateral migration matrices (Dańko et al. 2024; Kupiszewska and Wiśniowski, 2009). Third, the period under consideration offers annual flow data over two decades with stable national borders, allowing for consistent longitudinal and cross-country comparisons of migration patterns. This is in contrast to other migration cases, such as internal migration, where data may be less frequent and geographical units change over time (Lomax, 2022). Finally, intra-European migration has been a major testing ground for macro-level theories, particularly the gravity model (Ramos and Suriñach, 2017) and MST (DeWaard et al., 2012), which provide the two driver specifications evaluated in this application.

### Data

To study intra-European migration, I use yearly, country-to-country flow data developed by Dańko et al. (2024) as part of the Human Migration Database (HMigD; Dańko 2023). Annual bilateral flows are particularly valuable for capturing temporal variation, including policy changes and economic shocks, which are highly salient during the period under consideration (European Commission, 2015; Vatta, 2017). A significant advantage of the HMigD is that it corrects for misreporting and undercounting by integrating multiple data sources: Eurostat (2023a; 2023b; ), United Nations Department of Economic and Social Affairs (UNDESA, 2015), the German Federal Statistical Office (DESIS, 2023), the UK’s Office for National Statistics (ONS, 2020), and the IMEM and MIMOSA projects (IMEM, 2012; Kupiszewska and Wiśniowski, 2009; Raymer et al., 2013; Van Der Erf, 2009IMEM). These sources are harmonised using a Bayesian modelling framework that incorporates bilateral flow data, metadata on registration systems, and expert assessments of data reliability, adjusting for definitional inconsistencies (eg who qualifies as an international migrant) and heterogeneous reporting practices across statistical systems (Dańko et al., 2024).

**Fig. 2:**
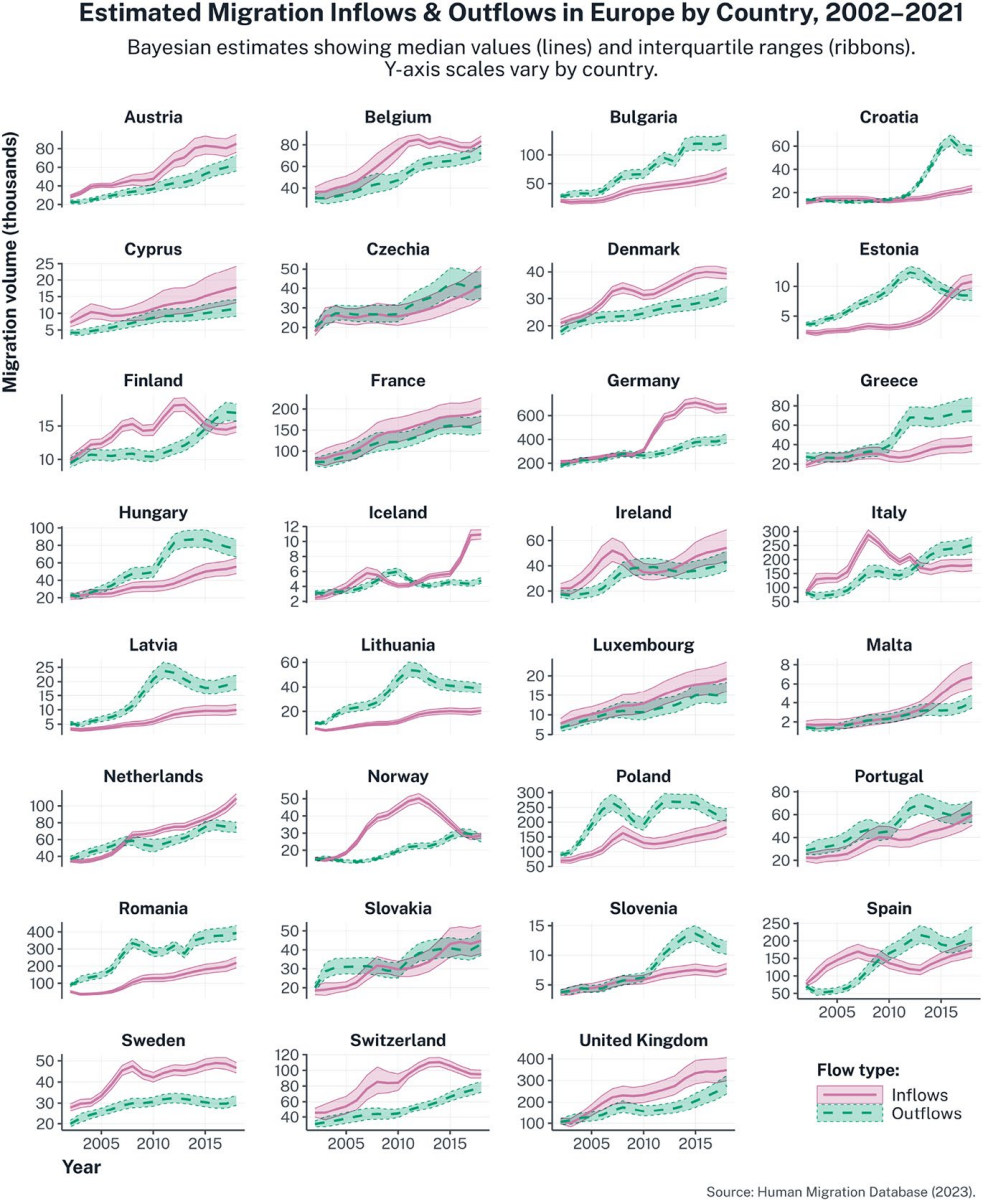
HMigD estimates of aggregate migration inflows (in pink) and outflows (in green) in 31 European countries between 2002 and 2021. Posterior medians (lines) and interquartile ranges (ribbons) from the Bayesian estimation process are reported, revealing substantial cross-country heterogeneity in the volume and evolution of intra-European migration flows.

HMigD estimates of migration inflows and outflows for each country in the sample between 2002 and 2021 are shown in Fig. 2. Each panel represents a country, with inflows shown as pink solid lines and outflows as green dashed lines; shaded ribbons denote interquartile uncertainty ranges derived from the Bayesian estimation process. The varying y-axis scales reflect substantial heterogeneity in migration volumes across the 31 countries. The figure illustrates the magnitude of intra-European mobility and its general upward trend, particularly in Western European countries, where inflows consistently exceed outflows. In contrast, many Eastern European countries experience persistently higher outflows, indicative of sustained emigration. Finally, countries such as Italy and Spain display fluctuations consistent with economic cycles, policy shifts, and differences in registration systems (Dańko et al., 2024).

### Theory Operationalisation

In line with the theoretical discussion in Sect. 2, gravity and MST are evaluated by translating them into sets of bilateral predictors explaining yearly flows between all country pairs from 2002 to 2021. The dependent variable is the estimated number of migrants moving from country *i* to country *j* in year *t*, taken from the HMigD. Because the dataset forms a complete bilateral panel and the focus is on macro-level drivers, migration flows are modelled using PPML regressions with origin, destination, and year fixed effects. The PPML estimator is widely used in migration research due to its robustness to heteroskedasticity, ability to handle zero-valued flows, and consistency under correct mean specification (Poot et al. 2016; Santos Silva and Tenreyro, 2006, 2022; Weidner and Zylkin, 2021). Standard errors are three-way clustered by origin, destination, and year to account for correlated shocks and unobserved heterogeneity (Pfaffermayr, 2023).

As a point of reference, a baseline model is estimated including only the three fixed effects. Its explanatory power provides a benchmark against which the added contribution of gravity and MST predictors can be assessed. The gravity and MST specifications follow the empirical applications of Ramos and Suriñach (2017) and DeWaard et al. (2012). The gravity model includes as predictors the bilateral geographic distance between country capitals and the lagged ratio of origin and destination population sizes, which remain identifiable despite fixed effects; country-specific population sizes are not considered as they are fully collinear with origin and destination fixed effects. The MST specification extends the gravity model by including further bilateral linkages: contiguity, shared language, the lagged ratio of GDP per capita between origin and destination, migrant stocks in the year 2000 (proxying network and cumulative causation effects), joint membership in the former Eastern Bloc (proxying shared geopolitical history), and lagged joint membership in the EU (proxying institutional integration). With the exception of the lagged population and GDP ratios and EU membership, all predictors are time invariant. Predictor variables and their sources are described in Appendix A.

Although several simulation strategies could in principle be used for Steps 2–4 of the procedure, this application uses macro-level simulations as described in Sect. 3.2. This choice reflects both theoretical and computational considerations. Gravity and MST are macro-structural theories centred on bilateral linkages rather than micro-level behaviour, making a system-level simulation more appropriate. Moreover, the scale of European migration, which exceeded three million movers in 2021 alone, would render micro-level simulation approaches computationally prohibitive. Hence, macro-level simulations represent an efficient and theoretically coherent strategy of generating theory-guided counterfactual migration systems.

### Step 1: Regression Results

The PPML regression estimates for the baseline, gravity, and MST models are reported in Table 1, showing clear differences between the two theoretical specifications^3^. In the gravity model, corridors with a 10% greater geographic distance experience migration flows that are approximately 8% lower, while the coefficient of lagged population ratio is not significant. In the MST model, the distance coefficient is no longer significant; neither are factors such as lagged population ratio, contiguity, lagged GDP ratio, and lagged EU membership. Instead, the MST model highlights socio-historical linkages: migration flows between countries that share a common official language are estimated to be about 67% larger than flows between otherwise comparable country pairs that do not share a common official language, while migration flows between countries with a shared geopolitical past are estimated to be about 169% larger than flows between countries without such a shared history. Processes of cumulative causation also appear relevant in the MST model: holding everything else constant, a 10% increase in bilateral migrant stocks in the year 2000 is associated with a 3.9% increase in subsequent migration flows between two countries.

To assess the PPML results, the Ramsey Regression Equation Specification Error Test (RESET) was performed to detect potential model misspecification, as recommended by Santos Silva and Tenreyro (2006). All models except the baseline pass the RESET with *p* values $>0.10$, suggesting correct model specification. Because PPML models are non-linear, a standard interpretation of the $R^2$ as explained variance is not possible: hence, squared correlations between fitted and observed values and an adjusted pseudo-$R^2$ were computed following Santos Silva and Tenreyro (2006) and Allison (2014). For the gravity and MST models, the adjusted pseudo-$R^2$ was computed also within origin–destination-year clusters (within adjusted pseudo-$R^2$). Values of these goodness-of-fit statistics show large improvements from the baseline model as more predictors are introduced. With only two predictors, the gravity model attains a squared correlation of 0.59 and an adjusted pseudo-$R^2$ of 0.77, consistent with previous findings on high predictive performance in gravity models of migration (Termote, 2002). The MST performs even better, attaining a squared correlation of 0.65 and an adjusted pseudo-$R^2$ of 0.82.

Because the dependent variable stems from Bayesian estimation, the PPML models in Table 1 use the posterior means of bilateral flows. To assess the sensitivity of the results to estimate uncertainty, all models were re-estimated using alternative dependent variables drawn from different posterior quantiles and exhibit no substantive changes (see Appendix B, Table 2, Table 3 and Table 4). In addition, to address potential concerns that some bilateral predictors, such as geographic distance, may have been included in the HMigD estimation process and potentially inflate the explanatory power of the PPML models, all regressions were replicated on a subsample of corridor-year observations using Eurostat migration flow data as the dependent variable (Eurostat, 2023b). The results of this replication appear consistent with the HMigD models’ explanatory power, indicating that high performance is not driven by dependence between the dependent variable and the predictors (see Appendix C, Fig. 6 and Table 5). Importantly, any undetected correlations between predictors and estimated flows would bias the subsequent simulations toward resembling the observed system, making the spatial evaluation in Steps 2–4 conservative rather than overstated. Finally, as the period under study includes the COVID-19 pandemic, which has been found to have severely disrupted both internal and international migration flows worldwide (González-Leonardo et al., 2022; Klein et al., 2024; Lerpold et al., 2023), the PPML regressions were replicated excluding the years 2019–2021: this replication shows no substantial changes in explanatory power for either theory and suggests that this period did not significantly impair the models’ performance (see Appendix D, Table 6).

**Table 1:** Step 1: results of PPML regressions using different explanatory models

Model	Baseline	Gravity	MST
*Predictor variables*			
Distance (log)		−0.7955$$^{***}$$	−0.1959
		(0.1145)	(0.1266)
Population ratio (lag, log)		−0.0454	0.0930
		(0.2756)	(0.2845)
Contiguity			−0.1630
			(0.1538)
Common official language			0.5117$^{*}$
			(0.1996)
GDP ratio (lag, log)			0.4369
			(0.6576)
Migrant stock (log)			0.3893$$^{***}$$
			(0.0578)
Shared geopolitical history			0.9911$^{*}$
			(0.3918)
Shared EU membership (lag)			0.0034
			(0.1251)
*Fixed effects*			
Origin country	$\checkmark$	$\checkmark$	$\checkmark$
Destination country	$\checkmark$	$\checkmark$	$\checkmark$
Year	$\checkmark$	$\checkmark$	$\checkmark$
*Fit statistics*			
Observations	18,600	18,600	18,600
Squared correlation	0.52252	0.58673	0.64782
Adjusted pseudo-R$^2$	0.72398	0.76556	0.82276
Within adjusted pseudo-R$^2$		0.15064	0.35789
Ramsey RESET	0.0334	0.1860	0.2032

Standard errors (in parentheses) clustered by origin, destination, and year.

Adjusted pseudo-R$^2$ is the McFadden’s R $^2$. RESET *p* values computed following Santos Silva and Tenreyo (2006).

Significance codes: $$\dagger $$: 0.1, *: 0.05, **: 0.01, ***: 0.001

### Steps 2–4: Simulation Results

The fitted values from the PPML regressions in Table 1 serve as inputs for Steps 2–4 of the proposed method. Following the simulation strategy outlined in Sect. 3.2, 10,000 synthetic migration systems are generated for each theory, gravity and MST, and for each year of the panel. In every simulated system, migration flows are determined exclusively by the predictors included in the corresponding PPML specification. The resulting set of simulations provides a distribution of counterfactual migration systems against which the observed system can be evaluated.

Steps 3 and 4 implement the spatial assessment at increasingly finer levels of resolution, starting with the system level using migration indices, then the country level using Mahalanobis distance, and finally the corridor level in four edge cases.

#### System-Level Evaluation: Migration Indices

**Fig. 3:**
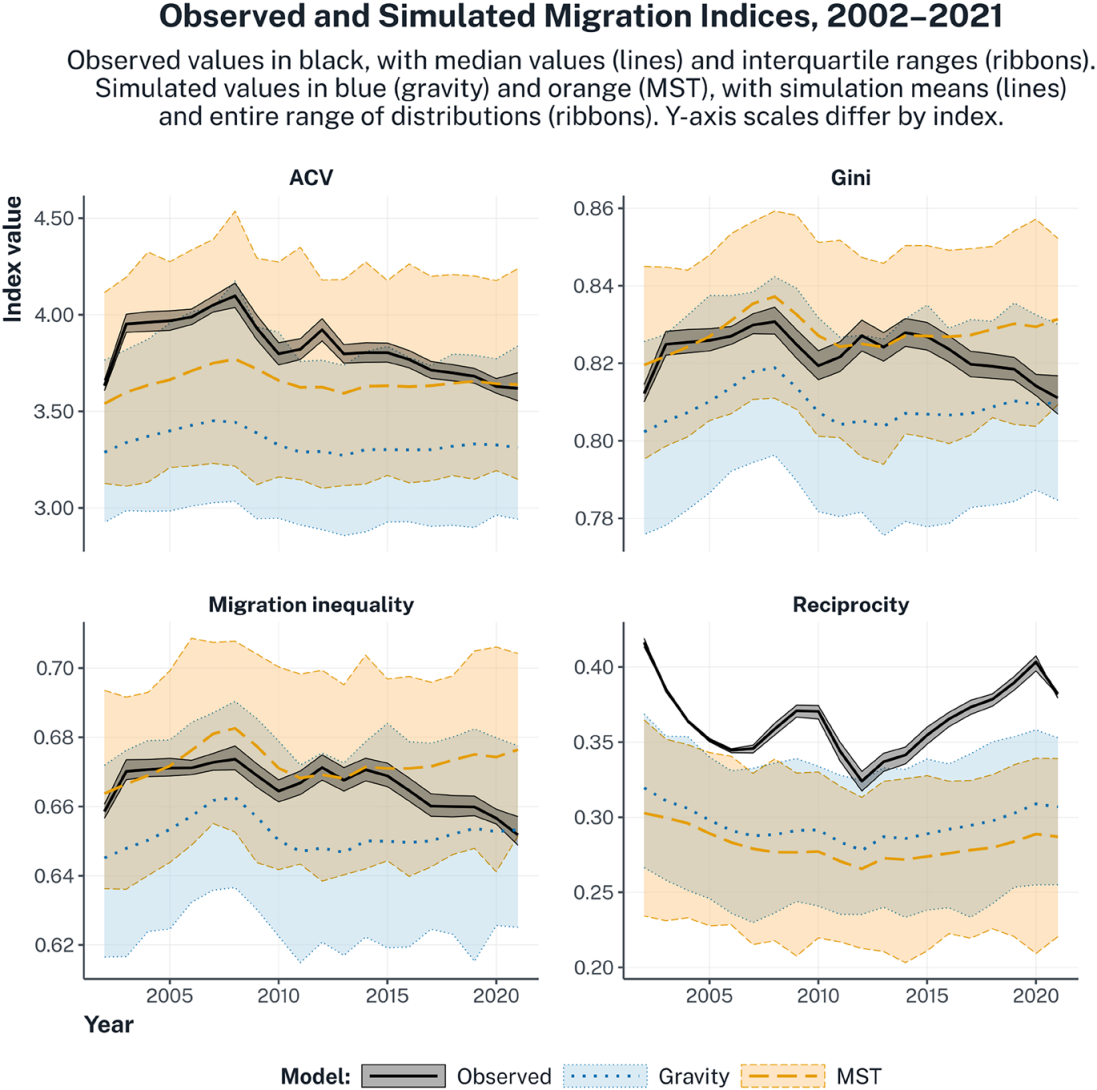
Accuracy of two explanatory models in accounting for the aggregate spatial features of the intra-European migration system over time. Spatial features measured using Aggregate Coefficient of Variation (ACV), Gini, migration inequality, and reciprocity. Observed values are reported in black, with posterior medians as solid lines and interquartile ranges as shaded ribbons. The full outcome ranges, generated by 10,000 simulations per year and model and , propagating the 95% regression uncertainty in the migration flows estimated in Table 1, are reported as shaded ribbons, in blue for gravity and orange for MST, with simulation means as dotted and dashed lines, respectively. Overlaps between the observed value and the simulation distributions indicate that the model can reproduce the observed pattern given regression uncertainty.

The first level of assessment examines whether theoretical models reproduce aggregate spatial features of the migration system. Figure 3 illustrates this for four migration indices: the ACV, the Gini Index, the Migration Inequality Index, and the Reciprocity Index. For each year, the distributions of simulated index values are shown for gravity (blue) and MST (orange), while the observed values appear as black solid lines with interquartile uncertainty ribbons. Mean values of the simulations are marked with dotted (gravity) and dashed (MST) lines to facilitate comparison. Because uncertainty propagates from the PPML fitted values to the simulations, overlaps between simulated and observed values indicate that the model could reproduce a given spatial feature given 95% regression uncertainty.

The results reveal systematic patterns. For migration concentration, both gravity and MST reproduce the observed levels reasonably well, albeit with some differences across indices. Gravity simulations align closely with the observed Gini Index and Migration Inequality Index, indicating that a parsimonious specification based on geographic distance and relative population size is sufficient to capture much of the overall concentration of intra-European migration. However, gravity underpredicts the ACV in some years, suggesting that the model struggles in capturing the influence of large, dominant corridors, to which the ACV is more sensitive. The MST model performs marginally better across all concentration measures and is the only specification that consistently reproduces observed values for all three indices.

Moving to reciprocity, we observe a different pattern. The observed reciprocity values are very high, surpassing 0.40 in several years (2002, 2003, 2020), meaning that, in those years, over 40% of migrants were moving in corridors where flows occur bilaterally. Yet, the two theories predict a much more asymmetric structure, failing to reproduce the observed reciprocity in all years except 2012 and 2013, where the range of gravity includes the observed reciprocity value. Overall, gravity appears to perform better than MST in reciprocity, as its simulation range covers slightly higher values.

#### Country-Level Evaluation: Mahalanobis Distance in Inflows

**Fig. 4:**
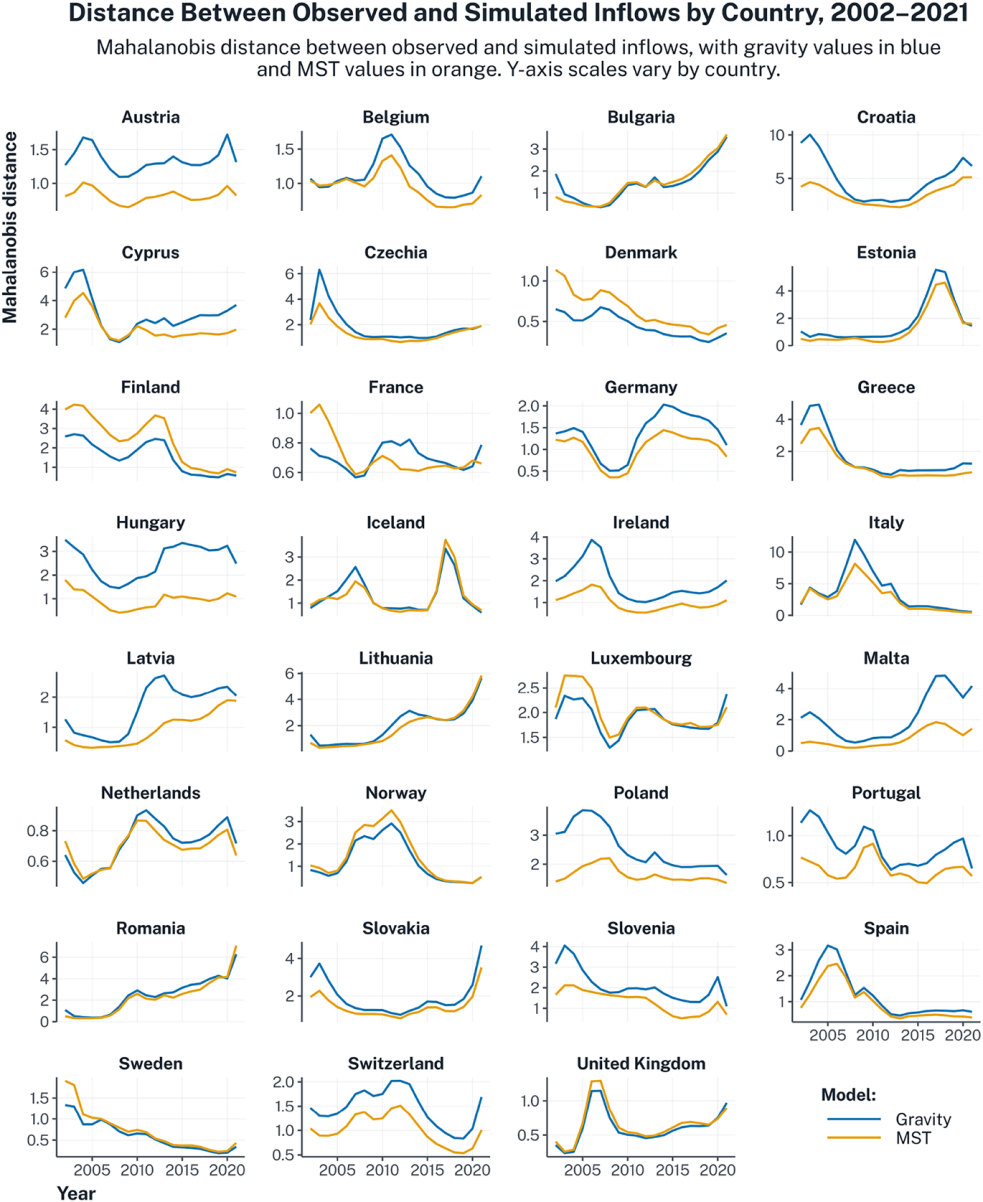
Distance between observed and simulated inflows in the intra-European migration system by country and over time. Mahalanobis distances were computed by extracting vectors of inflows for each country and year from the observed migration system, and comparing these vectors with 10,000 equivalent vectors stemming from the model-based simulations. This process was conducted separately for the two models, gravity (blue) and MST (orange). Lower Mahalanobis distance values indicate a closer match between simulated and observed inflows.

Moving to the intermediate spatial scale, Fig. 4 reports Mahalanobis distances between observed and simulated inflows for each country and year, separately for gravity (blue) and MST (orange). This measure captures how unusual each country’s observed inflow profile is relative to what each model predicts.

Several insights emerge. First, MST generally achieves better performance (ie lower distances) than gravity for most countries, in particular for Austria, Germany, Hungary, Ireland, Latvia, Poland, Portugal, and Switzerland. Second, gravity performs relatively better for the Nordic countries (Denmark, Finland, Norway, and Sweden), France pre-2008, the Netherlands pre-2008, and Luxembourg pre-2010. Third, the magnitude of Mahalanobis distances reveals where and when flows are hardest to predict, regardless of theory. For countries often characterised as peripheral within the European migration system (eg Croatia, Cyprus, Czechia, Estonia, Finland, Greece, Lithuania, Malta, Romania, Slovakia, and Slovenia), observed flows tend to lie far from both sets of simulations, as evidenced by the larger Mahalanobis distance values ($D^2 > 4.00$). Further, some countries exhibit sharp spikes over specific periods, such as Estonia in 2016–2017, Iceland in 2017, Lithuania, Romania, and Slovakia during the years affected by the COVID-19 pandemic (2020–2021), and Italy between 2007 and 2014, suggesting the influence of unmodelled shocks beyond what could be controlled for by the country and time fixed effects.

#### Corridor-Level Evaluation

**Fig. 5:**
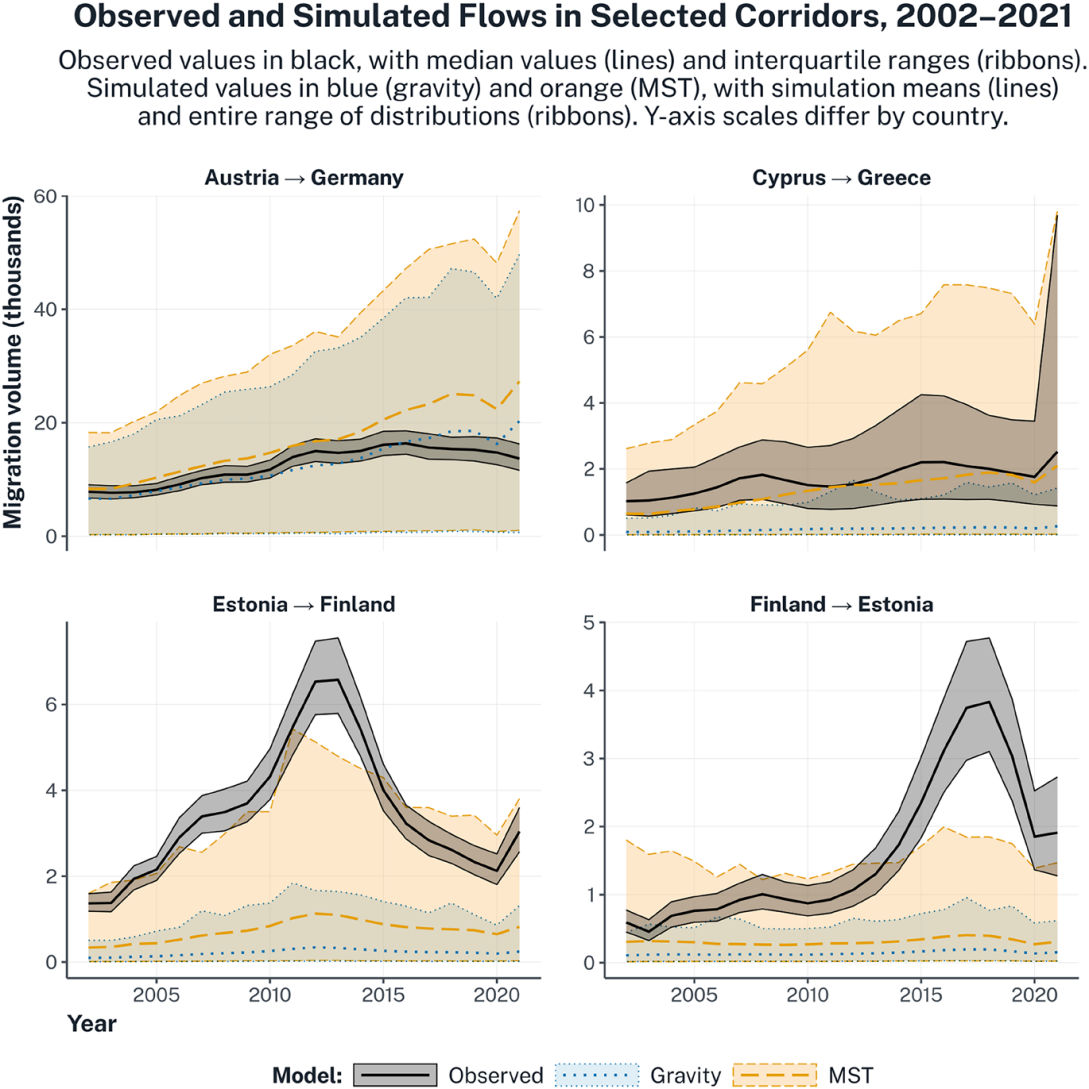
Accuracy of two explanatory models in accounting for the migration flows occurring in four exemplary corridors over time. For each of the four corridors, observed values are reported in black, with posterior medians (solid lines) and interquartile ranges (shaded ribbons). The full outcome ranges, generated by 10,000 simulations per corridor, year, and model and, propagating the 95% regression uncertainty in the migration flows estimated in Table 1, are reported as shaded ribbons, in blue for gravity and orange for MST, with simulation means as dotted and dashed lines, respectively. Overlaps between the observed value and the simulation distributions indicate that the model can reproduce the observed pattern given regression uncertainty. The four corridors were selected as illustrative contrasts: (1) flows in the first corridor, Austria to Germany, are well reproduced by both models; (2) flows in the second corridor, Cyprus to Greece, are well reproduced by MST only; (3–4) flows in the last two corridors, Estonia to Finland and Finland to Estonia, are not well reproduced by either model.

**Fig. 6:**
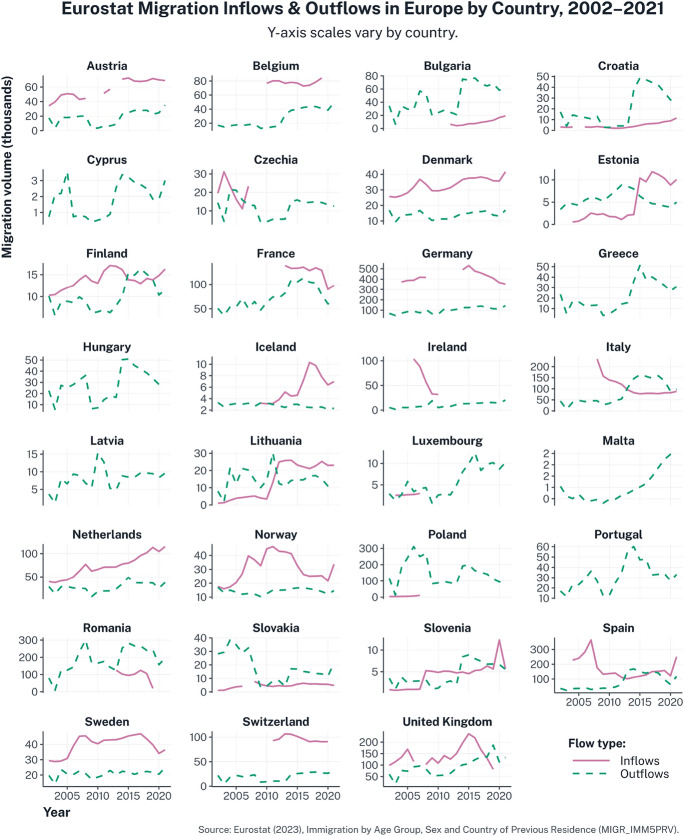
Eurostat estimates of aggregate migration inflows (in pink) and outflows (in green) in 31 European countries between 2002 and 2021.

Finally, at the most granular spatial level, Fig. 5 examines the spatial accuracy of the two theories for four example corridors: Austria to Germany, Cyprus to Greece, Estonia to Finland, and Finland to Estonia. In the plot, observed flows over time are reported as black solid lines with interquartile uncertainty ribbons. The full range of simulated flows are reported as ribbons, in blue for gravity and orange for MST, with simulation means as dotted (gravity) and dashed lines (MST). Overlaps between the observed value and the simulated distribution indicate that the observed flow volume is consistent with what the theory would generate, given the regression uncertainty of 95%.

The four corridors were chosen as they represent edge cases that best illustrate when and where different theoretical frameworks may be appropriate. In particular, in the corridor Austria to Germany, which involves two contiguous EU countries that share a common official language, both gravity and MST appear to perform well, as the observed flows fall within the range of simulated values. Conversely, in the case of Cyprus to Greece, which involves two countries relatively distant geographically but connected by historical ties and a common language, only the MST model performs well, as the gravity simulations only seldom touch the observed range. Finally, the two reciprocal corridors, Estonia to Finland and Finland to Estonia, could not be easily replicated by either theory, even though MST achieves an overall better performance for both. Both corridors exhibit sudden spikes in the migration volume in the periods 2010–2015 (Estonia to Finland) and 2015–2020 (Finland to Estonia), which could not be accounted for even when including the gravity and MST predictors and fixed effects, reflecting the country-level findings presented in Fig. 4.

**Fig. 7:**
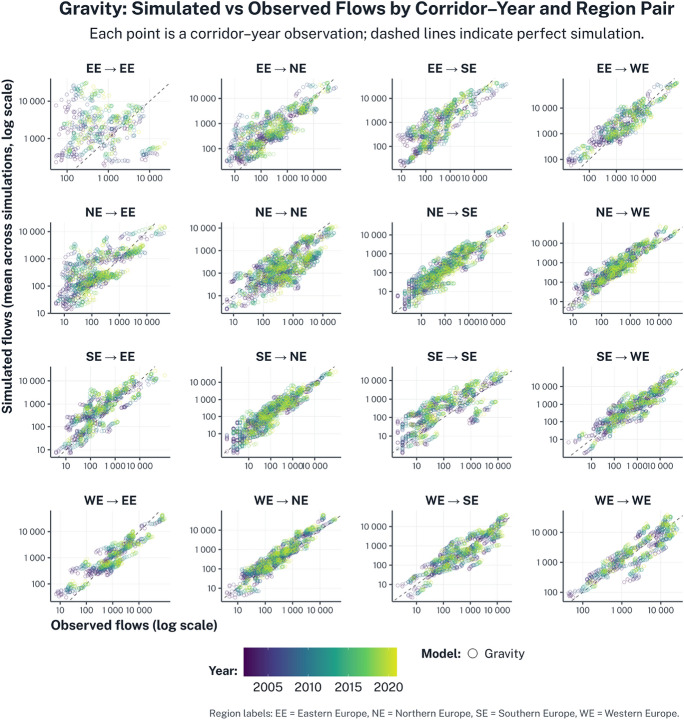
Comparison of the gravity-simulated and observed migration flows by corridor, year, and region. For each corridor–year combination, the points compare the observed flows with the mean of 10,000 simulated flows under the gravity model (circles). The diagonal indicates perfect simulation: simulated flows are equal to the observed ones. The scatterplots are grouped by the regions to which the 31 countries belong, as origins or destinations: Eastern Europe (EE), Northern Europe (NE), Southern Europe (SE), and Western Europe (WE).

**Fig. 8:**
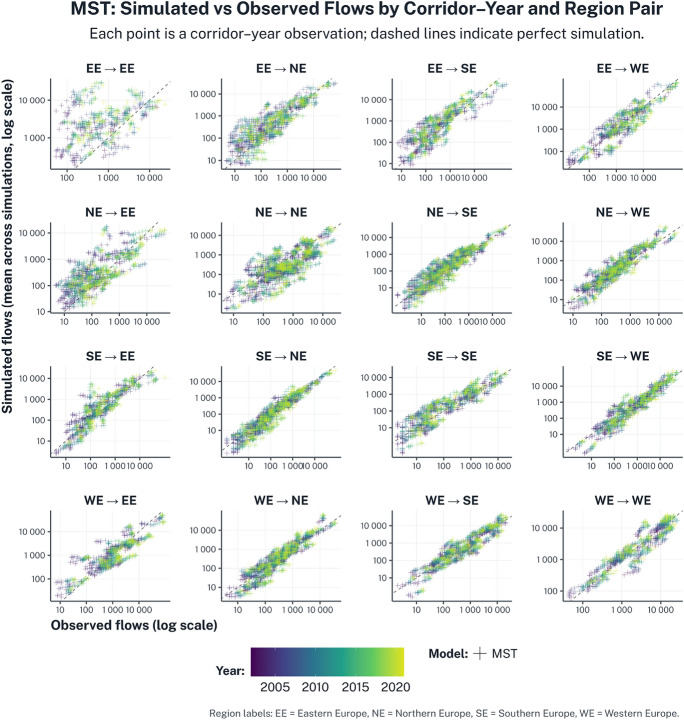
Comparison of the MST-simulated and observed migration flows by corridor, year, and region. For each corridor–year combination, the points compare the observed flows with the mean of 10,000 simulated flows under the MST model (crosses). The diagonal indicates perfect simulation: the simulated flows are equal to the observed ones. The scatterplots are grouped by the regions to which the 31 countries belong, as origins or destinations: Eastern Europe (EE), Northern Europe (NE), Southern Europe (SE), and Western Europe (WE).

As a complementary diagnostic, Appendix E reports the observed and simulated (mean) flow values for all corridor–year observations, separately for gravity (Fig. 7) and MST (Fig. 8). The scatterplots are faceted by the regions to which the origin and destination countries belong according to the United Nations geoscheme (United Nations Statistics Division, 2024), with the country–region correspondence reported in Table 7. Two main patterns emerge. First, there is no evidence of systematic temporal drift in model performance: deviations from the diagonal, which denotes perfect agreement between simulated and observed flow values, remain broadly stable across years, indicating that neither model improves nor deteriorates markedly over time. Second, while MST performs slightly better than gravity for several regional corridors, the relative performance of the two models is otherwise heterogeneous and does not follow a consistent regional hierarchy. One pattern is particularly robust across both models: migration corridors connecting two Eastern European countries, two Northern European countries, and from Northern to Eastern European countries are systematically poorly predicted, with the two theories achieving the lowest spatial accuracy.

## Discussion

This article set out to evaluate migration theories not only by their explanatory power, but also by their ability to reproduce the spatial organisation of migration flows. Applying a novel simulation-based spatial test to intra-European migration between 2002 and 2021 yields three central findings. First, both the gravity model and MST achieve strong explanatory power in conventional regression terms, with MST outperforming gravity according to standard goodness-of-fit measures. Second, when evaluated spatially, both theories fall short of reproducing important spatial features of the European migration system, most notably the observed levels of reciprocity in flows. Third, these shortcomings are not uniform across time and space: model performance varies substantially across system-level patterns, country inflows, and specific migration corridors.

The core theoretical contribution of this article lies in demonstrating that explanatory power and spatial accuracy constitute analytically distinct dimensions of theory-testing. In this application, MST explains a larger share of variance in bilateral flows than gravity, yet both theories generate simulated migration systems that diverge from the observed one in fundamental ways. Reliance on regression-based goodness-of-fit measures alone would therefore have supported a straightforward conclusion that MST provides the superior explanation of intra-European migration. The spatial evaluation shows that such a conclusion would be incomplete: despite strong explanatory power, both theories systematically misrepresent how migration is organised across space and over time. As migration patterns are the empirical imprints of underlying mechanisms (DeWaard and Ha, 2019), these misfits are not minor deviations, but signal that the theories differ in important ways from the pattern-generating processes of the observed system.

Building on the regression results, the simulation-based analysis evaluates spatial accuracy at increasingly fine levels of spatial resolution. At the system level, the two theories exhibit nuanced but distinct patterns of spatial fit. For migration concentration, both theories perform well overall, with the MST model performing marginally better across all concentration measures. By contrast, for reciprocity, both theories fail to match the high levels observed in most years, though gravity performs relatively better than MST, whose simulations imply more asymmetric exchange patterns. This reciprocity misfit should be interpreted in light of Europe’s distinctive institutional setting. Freedom of movement within the EU, complemented by bilateral mobility agreements with non-EU countries such as Switzerland, creates unusually symmetric conditions for cross-border mobility across a large set of countries, which facilitate not only permanent relocation but also short-term, temporary, and circular forms of migration. Such forms of mobility are common in labour migration and international student mobility and can generate high levels of reciprocal exchange even when long-term settlement remains uneven. These dynamics are prevalent in Europe, but only weakly theorised by gravity and MST, helping to explain why both models systematically underestimate reciprocity at the system level.

Moving to the country level, the spatial tests reveal further heterogeneity in model performance across countries and time. Several countries exhibit particularly volatile time trends, as indicated by the spikes, pointing to the interference of sudden shocks. An example of this is the COVID-19 pandemic, which affected the gravity and MST performance for Lithuania, Romania, and Slovakia. Given the extraordinary policy interventions that were implemented during this period, curtailing both internal and international migration, this period can be understood as a stress test for the models, where misfits likely reflect unpredictable policy shocks, rather than theoretical shortcomings.

Looking at other cases, MST generally outperforms gravity across much of Central Europe, where historical, geopolitical, and institutional linkages appear to play a stronger role in shaping migration flows. At the same time, MST appears to perform poorly in Northern Europe. This discrepancy highlights the importance of evaluating the spatial expectations of different theoretical frameworks, as MST tends to emphasise hierarchical core–periphery relations rooted in unequal political and economic ties. While such mechanisms are highly relevant at the global scale, Europe as a whole can be seen as a global migration ‘core’, but within Europe, and especially within Northern Europe, migration is organised more through multidirectional exchanges. Further, Nordic countries exhibit distinctive institutional and social features, such as extensive welfare systems, regulated labour markets, and high levels of institutional integration, that set them apart from other regional clusters and are not explicitly incorporated into either gravity or MST.

Theoretical shortcomings become visible also at the corridor level. A striking example is provided by the reciprocal migration pair connecting Estonia and Finland. Both gravity and MST fail to reproduce the sharp increase in migration from Estonia to Finland between 2010 and 2015, followed by a subsequent rise in migration in the opposite direction between 2015 and 2020, as evidenced also by the lower performance of the two theories in the country-level analysis. Existing research links the Estonia-Finland pattern to post-enlargement labour mobility, student migration, and later return or circular migration facilitated by geographic proximity and institutional integration (Anniste and Tammaru, 2014; Jakobson et al., 2012; Toomistu et al., 2024). While such dynamics of return and circular migration naturally generate high reciprocity at the systemic and corridor level, they are not explicitly captured by either theory. More generally, the diagnostic scatterplots show that corridors within certain regions, in particular within Eastern Europe, within Northern Europe, and from Northern to Eastern Europe, are systematically poorly reproduced by both models. This shared misfit points to common theoretical blind spots in accounting for the specific regional patterns of intra-European migration.

The present empirical application cannot directly test the role of return and circular migration in shaping intra-European flows, as it relies on aggregate bilateral data that do not distinguish between first-time moves, return migration, or repeated cross-border mobility. As a result, it is not possible here to isolate the specific life-course mechanisms (such as temporary labour migration followed by return) that would be required to adjudicate between competing explanations of reciprocity at the corridor level. Importantly, this limitation pertains to the data and simulation design used in this application, rather than to the proposed method itself. As outlined in Sect. 3, the same procedure could be implemented using individual-level data and micro-level simulation strategies to evaluate theories that explicitly operate at lower levels of analysis, such as network theory or the new economics of labour migration.

A further limitation of the application concerns its geographical scope. By focusing exclusively on intra-European international migration, the analysis excludes some of the world’s most prominent migration corridors, including South–North migration flows to Europe, North America, and the Gulf states. These systems are characterised by stronger asymmetries in income, legal status, and rights, conditions under which both gravity and MST may perform differently. One of the main aims of the proposed method is precisely to make the spatial assumptions of competing migration theories explicit; as such, the performance of theories is expected to vary across contexts. Applying the framework beyond Europe would allow researchers to assess where theories such as MST perform better in explaining asymmetric, historically structured systems, and where parsimonious spatial frictions captured by gravity remain sufficient. Similarly, future applications may consider internal migration cases to demonstrate the method’s portability and evaluate relevant theories in other settings.

More broadly, the results of the intra-European application underscore the value of treating migration systems as spatial objects that theories should be able to reproduce. The intended contribution of this article is not to adjudicate definitively between gravity and MST, nor between specific operationalisations of these theories, but to demonstrate how spatial theory testing can reveal shortcomings that remain invisible to conventional model evaluation. Importantly, the proposed framework is agnostic to specific theories or spatial indices and can be adapted to test alternative theoretical mechanisms across diverse migration contexts. By making spatial accuracy an explicit criterion of theory testing, this approach complements regression-based methods and enables more transparent, comparative, and spatially aware evaluations of migration processes.

## Figures and Tables

**Fig. 9:**
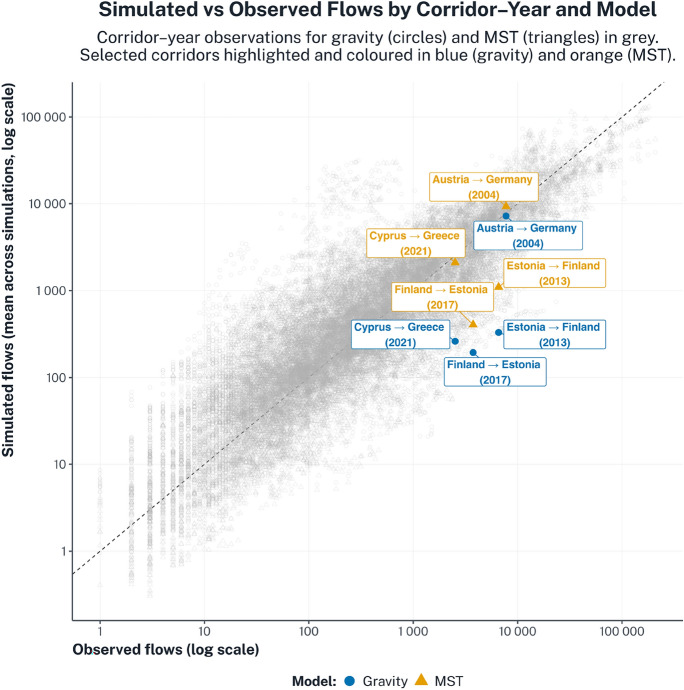
Comparison of simulated and observed migration flows by corridor, year, and model. For each corridor–year combination, the points compare the observed flows with the mean of 10,000 simulated flows under two explanatory models, gravity (dots, highlighted in blue) and MST (triangles, highlighted in orange). The diagonal indicates perfect simulation: the simulated flows equal the observed ones. Four corridor-year combinations are highlighted: (1) flows from Austria to Germany in 2004; (2) flows from Cyprus to Greece in 2021; (3) flows from Estonia to Finland in 2013; and (4) flows from Finland to Estonia in 2017. These four cases were chosen to illustrate where and when simulated flows may deviate from the observed ones, illustrating model-specific strengths and weaknesses.

## Data Availability

As these data are produced and maintained by third parties, they cannot be uploaded directly to a private repository. However, the GitHub repository that accompanies the article, available at https://github.com/mmorellini/spatial-mig-tests, documents how each dataset can be accessed, along with all relevant links. Further, the GitHub repository includes the full set of processing steps, analysis scripts, and simulation code, enabling full replication of the regression models, simulations, and spatial evaluation metrics. The migration flow data used in this study are from the Human Migration Database (HMigD), compiled and harmonised by Dańko et al. (2024). These data are available for academic use and can be accessed through the HMigD online platform developed by Dańko (2023) at the following link: https://maciej-jan-danko.shinyapps.io/HMigD_Shiny_App_I. Additional variables used in the explanatory models are drawn from publicly available sources: (i) geographical distance (distance between capitals), contiguity, common official language, shared EU membership, and shared geopolitical history come from CEPII’s Gravity database (Conte et al., 2021); ii) GDP per capita as Purchasing Power Parity (PPP) in current international US dollars ($) is sourced from the World Bank (World Bank, 2025); iii) bilateral migrant stock in 2000 is sourced from the United Nations Department of Economic and Social Affairs (UNDESA, 2015, 2019).

## Appendix A: Data Availability Statement

The migration flow data used in this study are from the Human Migration Database (HMigD), compiled and harmonised by Dańko et al. (2024); Dańko (2023). These data are available for academic use and can be accessed through the HMigD online platform: https://maciej-jan-danko.shinyapps.io/HMigD_Shiny_App_I. Additional variables used in the explanatory models are drawn from publicly available sources:

(i) From the CEPII’s Gravity database (Conte et al., 2021): geographical distance, measured as distance between capitals (distcap); contiguity (contig); shared official language (comlang_off); shared geopolitical history (sibling_ever); shared EU membership (eu_both).
(ii) From the World Bank (World Bank, 2025): GDP per capita, Purchasing Power Parity (PPP) in current international US dollars ($; NY.GDP.PCAP.PP.CD).
(iii) From the United Nations Department of Economic and Social Affairs (UNDESA, 2015; UNDESA, 2019): bilateral migrant stock in the year 2000.

As these data are produced and maintained by third parties, they cannot be uploaded directly to a private repository. However,the GitHub repository that accompanies the article, available at https://github.com/mmorellini/spatial-mig-tests, documents how each dataset can be accessed,along with all relevant links. Further, the GitHub repository includes the full set of processingsteps, analysis scripts, and simulation code, enabling full replication of the regression models,simulations, and spatial evaluation metrics..

## Appendix B: PPML Regressions with Varying Dependent Variables

See Tables 2, 3, 4.

**Table 2:** PPML regressions of the baseline model with alternative dependent variables (migration flows). Alternative dependent variables were drawn from the HMigD posterior distribution (Dańko, 2023; Dańko et al., 2024) using different quantiles (q.)

Estimated migration flow	5^th^ q. (0.05)	25^th^ q. (0.25)	50^th^ q. (0.5)	75^th^ q. (0.75)	95^th^ q. (0.95)
*Predictor variables*					
None (baseline specification)					
*Fixed effects*					
Origin country	$\checkmark$	$\checkmark$	$\checkmark$	$\checkmark$	$\checkmark$
Destination country	$\checkmark$	$\checkmark$	$\checkmark$	$\checkmark$	$\checkmark$
Year	$\checkmark$	$\checkmark$	$\checkmark$	$\checkmark$	$\checkmark$
*Fit statistics*					
Observations	18,600	18,600	18,600	18,600	18,600
Squared correlation	0.55201	0.53452	0.52252	0.51068	0.49428
Adjusted pseudo-R$^2$	0.73536	0.72880	0.72398	0.71882	0.71117

Baseline specification includes only origin, destination, and year fixed effects.

Adjusted pseudo-R $^2$ is the McFadden’s R $^2$

**Table 3:** PPML regressions of the gravity model with alternative dependent variables (migration flows). Alternative dependent variables were drawn from the HMigD posterior distribution (Dańko, 2023; Dańko et al., 2024) using different quantiles (q.)

Estimated migration flow	5^th^ q. (0.05)	25^th^ q. (0.25)	50^th^ q. (0.5)	75^th^ q. (0.75)	95^th^ q. (0.95)
*Predictor variable*					
Distance (log)	−0.8286$$^{***}$$	−0.8095$$^{***}$$	−0.7955$$^{***}$$	−0.7812$$^{***}$$	−0.7608$$^{***}$$
	(0.1235)	(0.1180)	(0.1145)	(0.1114)	(0.1071)
Population ratio (lag, log)	−0.0629	−0.0503	−0.0454	−0.0430	−0.0431
	(0.2920)	(0.2831)	(0.2756)	(0.2671)	(0.2536)
*Fixed effects*					
Origin country	$\checkmark$	$\checkmark$	$\checkmark$	$\checkmark$	$\checkmark$
Destination country	$\checkmark$	$\checkmark$	$\checkmark$	$\checkmark$	$\checkmark$
Year	$\checkmark$	$\checkmark$	$\checkmark$	$\checkmark$	$\checkmark$
*Fit statistics*					
Observations	18,600	18,600	18,600	18,600	18,600
Squared correlation	0.61652	0.59943	0.58673	0.57345	0.55421
Adjusted pseudo-R$^2$	0.77594	0.77003	0.76556	0.76063	0.75315
Within Adjusted pseudo-R$^2$	0.15335	0.15204	0.15064	0.14871	0.14537

Standard errors (in parentheses) clustered by origin, destination, and year.

Adjusted pseudo-R $^2$ is the McFadden’s R $^2$. Significance codes: $\dagger$ : 0.1, *: 0.05, **: 0.01, ***: 0.001

**Table 4:** PPML regressions of the MST model with alternative dependent variables (migration flows). Alternative dependent variables were drawn from the HMigD posterior distribution (Dańko, 2023; Dańko et al., 2024) using different quantiles (q.)

Estimated migration flow	5^th^ q. (0.05)	25^th^ q. (0.25)	50^th^ q. (0.5)	75^th^ q. (0.75)	95^th^ q. (0.95)
*Predictor variables*					
Distance (log)	−0.2141$^{\dagger }$	−0.2035	−0.1959	−0.1883	−0.1772
	(0.1203)	(0.1240)	(0.1266)	(0.1292)	(0.1320)
Population ratio (lag, log)	0.0891	0.0944	0.0930	0.0881	0.0723
	(0.2897)	(0.2881)	(0.2845)	(0.2786)	(0.2655)
Contiguity	−0.1674	−0.1630	−0.1630	−0.1661	−0.1724
	(0.1527)	(0.1527)	(0.1538)	(0.1558)	(0.1599)
Common official language	0.4802$$^{**}$$	0.4967$$^{*}$$	0.5117$$^{*}$$	0.5301$$^{**}$$	0.5606$$^{**}$$
	(0.1843)	(0.1937)	(0.1996)	(0.2052)	(0.2109)
GDP ratio (lag, log)	0.5703	0.4926	0.4369	0.3800	0.3025
	(0.6727)	(0.6666)	(0.6576)	(0.6467)	(0.6220)
Migrant stock (log)	0.4054$$^{***}$$	0.3959$$^{***}$$	0.3893$$^{***}$$	0.3828$$^{***}$$	0.3734$$^{***}$$
	(0.0604)	(0.0589)	(0.0578)	(0.0569)	(0.0558)
Shared geopolitical history	0.8658$$^{*}$$	0.9419$$^{*}$$	0.9911$$^{*}$$	1.037$$^{**}$$	1.086$$^{**}$$
	(0.4072)	(0.3993)	(0.3918)	(0.3830)	(0.3660)
Shared EU membership (lag)	0.0208	0.0137	0.0034	−0.0115	−0.0421
	(0.1294)	(0.1257)	(0.1251)	(0.1280)	(0.1416)
*Fixed effects*					
Origin country	$\checkmark$	$\checkmark$	$\checkmark$	$\checkmark$	$\checkmark$
Destination country	$\checkmark$	$\checkmark$	$\checkmark$	$\checkmark$	$\checkmark$
Year	$\checkmark$	$\checkmark$	$\checkmark$	$\checkmark$	$\checkmark$
*Fit statistics*					
Observations	18,600	18,600	18,600	18,600	18,600
Squared correlation	0.67214	0.65837	0.64782	0.63658	0.61990
Adjusted pseudo-R$^2$	0.82812	0.82508	0.82276	0.82010	0.81574
Within adjusted pseudo-R$^2$	0.35052	0.35503	0.35789	0.36019	0.36208

Standard errors (in parentheses) clustered by origin, destination, and year.

*Adjusted pseudo-R* $^2$ is the McFadden’s R $^2$. Significance codes: $\dagger$: 0.1, *: 0.05, **: 0.01, ***: 0.001

## Appendix C: Replication with Eurostat data

See Table 5; Fig. 6.

**Table 5:** Results of PPML regressions using Eurostat data

Model	Baseline	Gravity	MST
*Predictor variables*			
Distance (log		−0.9431$$^{***}$$	−0.2878$$^{*}$$
		(0.1468)	(0.1394)
Population ratio (lag, log)		−0.2212	−0.0938
		(0.2451)	(0.1831)
Contiguity			−0.0065
			(0.1305)
Common official language			0.7156$$^{***}$$
			(0.1762)
GDP ratio (lag, log)			−1.829$$^{**}$$
			(0.6548)
Migrant stock (log)			0.3375$$^{***}$$
			(0.0616)
Shared geopolitical history			0.8608
			(0.5542)
Shared EU membership (lag)			0.0660
			(0.1973)
*Fixed effects*			
Origin country	$\checkmark$	$\checkmark$	$\checkmark$
Destination country	$\checkmark$	$\checkmark$	$\checkmark$
Year	$\checkmark$	$\checkmark$	$\checkmark$
*Fit statistics*			
Observations	10,364	10,364	10,364
Squared correlation	0.55603	0.63634	0.65660
Adjusted pseudo-R$^2$	0.74695	0.79818	0.83895
Within adjusted pseudo-R$^2$		0.20265	0.36375
Ramsey RESET	0.0046	0.2047	0.1910

Standard errors (in parentheses) clustered by origin, destination, and year.

Adjusted pseudo-R $^2$ is the McFadden’s R $^2$. RESET *p* values computed following Santos Silva and Tenreyro (2006). Signif. codes: $\dagger$: 0.1, *: 0.05, **: 0.01, ***: 0.001

## Appendix D: Replication with HMigD Subsample (Excluding 2019–2021)

See Table 6.

**Table 6:** Results of PPML regressions with HMigD data, excluding years affected by COVID-19 pandemic (2019–2021)

Model	Baseline	Gravity	MST
*Predictor variables*			
Distance (log)		−0.8418$$^{***}$$	−0.2452$$^{*}$$
		(0.1125)	(0.1168)
Population ratio (lag, log)		−0.0685	0.0434
		(0.2733)	(0.2665)
Contiguity			−0.1572
			(0.1567)
Common official language			0.5277$$^{**}$$
			(0.1917)
GDP ratio (lag, log)			1.166$^{\dagger }$
			(0.6272)
Migrant stock (log)			0.3980$$^{***}$$
			(0.0591)
Shared geopolitical history			0.8904$^{*}$
			(0.3650)
Shared EU membership (lag)			0.1260
			(0.1751)
*Fixed effects*			
Origin country	$\checkmark$	$\checkmark$	$\checkmark$
Destination country	$\checkmark$	$\checkmark$	$\checkmark$
Year	$\checkmark$	$\checkmark$	$\checkmark$
*Fit statistics*			
Observations	15,810	15,810	15,810
Squared correlation	0.52137	0.59950	0.66666
Adjusted pseudo-R$^2$	0.72198	0.76804	0.82780
Within adjusted pseudo-R$^2$		0.16568	0.38062
Ramsey RESET	0.0287	0.1584	0.1674

Standard errors (in parentheses) clustered by origin, destination, and year

Adjusted pseudo-R $^2$ is the McFadden’s R $^2$. RESET *p* values computed followingSantos Silva and Tenreyro (2006). Signif. codes: $\dagger$: 0.1, *: 0.05, **: 0.01, ***: 0.001

## Appendix E: Scatterplots with Observed vs Simulated Flows

See Figs. 7, 8, 9; Table 7.

**Table 7:** List of countries in the sample and corresponding ISO 3166-1 codes (alpha-2 and alpha-3) and UN region labels

Country	ISO alpha-2	ISO alpha-3	UN region
Austria	AT	AUT	Western Europe
Belgium	BE	BEL	Western Europe
Bulgaria	BG	BGR	Eastern Europe
Switzerland	CH	CHE	Western Europe
Cyprus	CY	CYP	Southern Europe
Czechia	CZ	CZE	Eastern Europe
Germany	DE	DEU	Western Europe
Denmark	DK	DNK	Northern Europe
Estonia	EE	EST	Northern Europe
Spain	ES	ESP	Southern Europe
Finland	FI	FIN	Northern Europe
France	FR	FRA	Western Europe
Greece	GR	GRC	Southern Europe
Croatia	HR	HRV	Southern Europe
Hungary	HU	HUN	Eastern Europe
Ireland	IE	IRL	Northern Europe
Iceland	IS	ISL	Northern Europe
Italy	IT	ITA	Southern Europe
Lithuania	LT	LTU	Northern Europe
Luxembourg	LU	LUX	Western Europe
Latvia	LV	LVA	Northern Europe
Malta	MT	MLT	Southern Europe
Netherlands	NL	NLD	Western Europe
Norway	NO	NOR	Northern Europe
Poland	PL	POL	Eastern Europe
Portugal	PT	PRT	Southern Europe
Romania	RO	ROU	Eastern Europe
Sweden	SE	SWE	Northern Europe
Slovenia	SI	SVN	Southern Europe
Slovakia	SK	SVK	Eastern Europe
United Kingdom	UK	GBR	Northern Europe

## Footnotes

1 Migration systems can also be represented as networks, where locations are ‘nodes’ and migration flows are the ‘edges’ connecting them (see for instance Akbari, 2021; Danchev and Porter, 2018; Huang and Butts 2023, 2024; Leal and Harder, 2021, 2023; McMillan, 2024; 2024Plotnikova and Ulceluse 2022; Tranos et al. 2015; Windzio, 2018).

2 The selected countries are (in alphabetical order): Austria, Belgium, Bulgaria, Croatia, Cyprus, Czechia, Denmark, Estonia, Finland, France, Germany, Greece, Hungary, Iceland, Ireland, Italy, Latvia, Lithuania, Luxembourg, Malta, the Netherlands, Norway, Poland, Portugal, Romania, Slovakia, Slovenia, Spain, Sweden, Switzerland, and the United Kingdom (UK).

3 Because PPML coefficients are semi-elasticities, log-transformed continuous predictors can be interpreted in percentage terms, while effects of binary predictors correspond to $(e^\beta - 1)\times 100$.
